# *Boikoetliso Ba Boko* (‘exercising the mind’): protocol for a mixed methods feasibility and acceptability study of a prototype mental health intervention for adolescents and young people with anxiety and depression

**DOI:** 10.3389/frcha.2025.1569135

**Published:** 2025-06-24

**Authors:** Claire Hart, Rachana Desai, Lauren Stuart, Shane A. Norris

**Affiliations:** ^1^ SAMRC Developmental Pathways for Health Research Unit, Department of Paediatrics, School of Clinical Medicine, Faculty of Health Sciences, University of the Witwatersrand, Johannesburg, South Africa; ^2^ School of Health and Human Development, University of Southampton, Southampton, United Kingdom

**Keywords:** adolescent mental health, feasibility study, acceptability, mixed methods, problem management plus, sub-Saharan Africa

## Abstract

**Background:**

Approximately one in four adolescents in Sub-Saharan Africa experiences significantly elevated symptoms of anxiety and depression. Those living in resource-constrained communities face heightened risks due to acute distress, trauma, and less responsive health care services. *Boikoetliso Ba Boko* (meaning ‘exercising the mind’) is an intervention prototype designed for adolescents and young people experiencing common mental health conditions.

**Methods:**

The prototype aims to implement a youth-centred community mental health intervention, through collaboration with existing public health systems and community resources. It seeks to enhance access to mental health support for adolescents and young people in Soweto, South Africa, by creating a dynamic and engaging therapeutic environment for those dealing with anxiety, depression, and suicidal ideation. This protocol outlines how we will evaluate the feasibility, acceptability, and implementation process of the prototype mental health intervention. We will enrol 200 at-risk adolescents and young people, aged 14–24 years, who will serve as their own controls, into the intervention and collect assessment and process evaluation data using mixed methods.

**Discussion:**

This study aims to provide a comprehensive understanding of how the *Boikoetliso Ba Boko* adolescent mental health prototype can be effectively evaluated and scaled up in resource-constrained communities. The findings will inform the development of a Phase II randomized controlled trial protocol to assess the prototype's efficacy.

**Ethics:**

Ethical approval was granted by the Human Ethics Research Committee of the University of the Witwatersrand (M231045 MED23-09-040). *Boikoetliso Ba Boko* is registered with the Pan African Clinical Trial Registry (PACTR202409702283764).

## Introduction

We have the largest generation of young people in history, with 1.8 billion individuals aged 10–24 years, comprising 40% of the global population (1). Adolescence is a critical developmental period for physical growth and brain development (2), as well as the formation of social capital, defined as the support, trust, and resources available through interpersonal relationships and community networks (3). Mental disorders are increasingly prevalent among adolescents (4), with over 70% of mental disorders onset before age 25, and more than half of young people experiencing at least one episode of mental ill-health by this age (5). Additionally, up to one-third of suicide attempts occur among adolescents (6). Despite this, adequate management and treatment services are often lacking in public health systems to effectively address adolescent mental health needs (7, 8). In this context, community-based resources, such as peer networks, schools, and local organizations, can be sources of social capital, offering accessible and context-specific support for adolescents experiencing mental health challenges.

In Sub-Saharan Africa depression and anxiety are the most common mental health conditions, affecting 27% and 30% of adolescents, respectively (9). Young people in resource-constrained communities face increased risks due to poverty, exposure to trauma, and social dysfunction, evidenced by persistent difficulties in engaging with expected social roles and relationships, such as those with family, peers, or school (10–12). These challenges can inhibit service engagement (13–15), and stigma further prevents adolescents from seeking help, even in times of significant distress (16–19). Given that symptoms typically emerge around age 15 and mental health disorders show a median persistence rate of 80%, there is an urgent need for accessible, context-sensitive interventions (20, 21). In Sub-Saharan Africa, community-based interventions have the potential to reduce barriers to care, address stigma, and strengthen adolescent mental health support systems (22).

While recognition of mental health needs is increasing, the global mental health response for adolescents remains inadequate, particularly in low- and middle-income countries, where the treatment gap exceeds 75% in some settings, including South Africa (23). Adolescents face a critical developmental window during which unaddressed mental health issues can negatively affect educational, social, and economic trajectories. Despite growing evidence that social support and connectedness are protective factors, interventions that strengthen adolescents’ existing community structures remain underdeveloped and underutilized (22). Community-based interventions, such as the Friendship Bench intervention in Zimbabwe, demonstrate that scalable, low-cost mental health care is achievable in a resource-constrained setting (24). A youth-focused adaptation of the program, the Youth Friendship Bench (YouFB), delivered by trained lay health workers, has shown compelling results, with participants experiencing more than a threefold reduction in depression symptoms, a fourfold reduction in anxiety symptoms, and a fivefold decrease in suicidal thoughts compared to those receiving standard care after a six-month follow-up (25–27).

The Lancet Psychiatry Commission on youth mental health advocates for the expansion of accessible, youth-centred, community-based services that are integrated into existing health systems and grounded in evidence-based practice (5). In response to this call, this paper presents the feasibility and acceptability protocol for *Boikoetliso Ba Boko* (B^3^), meaning ‘exercising the mind’ in Setswana, a context-specific intervention designed for adolescents at risk of anxiety, depression, and suicidal ideation. We hypothesize that B^3^ offers a pragmatic, scalable, and accessible solution that is youth-centred and aligned with the structural and social realities of resource-constrained settings. By adapting and implementing interventions like B^3^, we not only address a pressing public health gap but also strengthen support systems for adolescents and young people, contributing to their long-term mental health, development, and well-being.

## Methods

### Study design and setting

B^3^ is a Phase I intervention study of a youth-centred community mental health intervention conducted over 18 months. A mixed-method approach will evaluate B^3^, combining quantitative data from mental health assessments, process fidelity, and cost analysis with qualitative process evaluation data on feasibility and acceptability. In this study, feasibility refers to whether the intervention is successfully implemented within the specific setting, while acceptability focuses on how well the adolescent target population and other stakeholders (for example, caregivers, schools, or healthcare providers) accept and engage with the intervention. This study will be housed at the Developmental Pathways for Health Research Unit, located within Africa's largest hospital in Soweto, which has 35 years of experience in research and collaboration with the Soweto community. Soweto is an urban-poor area of the City of Johannesburg, covering 200 km^2^ with over 1.3 million people (6,400/km^2^), of which an estimated 450 000 are adolescents and young people (28). This study aims to identify and address specific needs, preferences, and barriers unique to this population, ensuring the intervention resonates with their lived experiences, is culturally appropriate, and contextually relevant.

### Study sample, recruitment, and eligibility criteria

We aim to enrol 200 adolescents (14–19 years) or young adults (20–24 years). Recruitment will be monitored closely, and where feasible, efforts will be made to maintain balance across age groups. However, achieving exact balance is not a formal requirement of the study design, and recruitment will proceed pragmatically within the target population. Participants will be recruited through established referral pathways from screening programs in Soweto that identify individuals at risk for depression, anxiety, or suicidal ideation using recognized screening tools: the Patient Health Questionnaire-9 (29), and Generalized Anxiety Disorder-7 (30) that are commonly used in this context (31). All referrals will be assessed by a social worker for eligibility. Eligible participants must: (i) reside in Soweto, (ii) be aged 14–24, (iii) score at risk for depression, anxiety, or suicidal thoughts on screening tools, and (iv) have a primary caregiver residing in the same household (for those under 18) who consents to their participation. Exclusions will apply to individuals with severe intellectual disabilities or communication difficulties, or those outside the age range. The inclusion and exclusion criteria can be found in Table 1 below.

**Table 1:** Inclusion and exclusion criteria.

Category	Criteria	Description	Assessment method
Inclusion	Adolescents/youth aged 14–24 years	Defined age range of participants	Self-report with confirmation via ID, birth certificate, or caregiver report
	Living in Soweto	Residence within Soweto, Johannesburg	Self-report and/or local address verification
	Primary caregiver of adolescent is resident in the same household	Caregiver resides in same dwelling and has primary caregiving responsibilities	Self-report from both caregiver and adolescent
	Scored at risk of probable depression and/or anxiety and/or suicide ideation on screening tools	Positive screen on recognized screening tools	GAD-7 (30) score ≥10; PHQ-9 (29) score ≥10; The ‘at risk’ category reflects moderate symptoms reported in the past 2 weeks, consistent with prior studies using these tools to signal probable caseness (29–31)
Exclusion	Severe intellectual disability or communication difficulties: The inability to provide informed consent or meaningfully participate in assessments due to cognitive or communicative impairments	Participants scoring above defined thresholds on cognitive understanding or communication domains will be excluded	WHO functional impairments questionnaire (36); WHO disability assessment schedule 2.0 (71)

### Ethics

The B^3^ prototype has ethical approval from the Human Ethics Research Committee of the University of the Witwatersrand (M231045 MED23-09-040) and is registered with the Pan African Clinical Trial Registry (PACTR202409702283764). Data collection will adhere to South African government legislation (POPIA) (32), and the study methods follow StaRI and SPIRIT guidelines for transparent reporting (33). Participants will provide assent or consent based on age: those 18 and older will give written consent, while those under 18 will require consent from a primary caregiver and their own written assent. Participants will be assured that their information will remain confidential, though complete anonymity cannot be guaranteed due to the in-person nature of the intervention prototype. All investigators are legally obligated to report suspected child abuse or neglect as per the Children's Act 38 of 2005 (34).

Given that participants are at risk for depression, anxiety, and suicidal ideation, specific guidelines will mitigate potential harm. A distress protocol will be implemented to address any concerns that arise during data collection or intervention sessions. The intervention team will be trained in these procedures, with regular supervision and continued training to ensure adherence. If a participant shows signs of significant distress, the session will be paused, and the participant will be given time and space to recover. The concern will be reported immediately to the study's social worker, who will conduct a preliminary assessment to determine the level of severity and urgency. For urgent cases requiring further clinical input, the social worker will consult the supervising psychologist. Referrals will be made promptly for participants requiring more specialized care, and they will be supported through the process of accessing appropriate healthcare professionals or longer-term services.

All incidents will be recorded and reviewed by the supervising psychologist within 24 hours. The intervention team will follow up to ensure that referrals are acted upon and that participants are receiving appropriate support. A risk register will be maintained throughout the study and reviewed regularly by the intervention team under the supervision of the social worker and psychologist. This will document emerging risks, including participant distress, recruitment challenges, or operational issues, and inform any necessary protocol adaptations to ensure the study's integrity and feasibility.

### Prototype development

**Figure 1:**
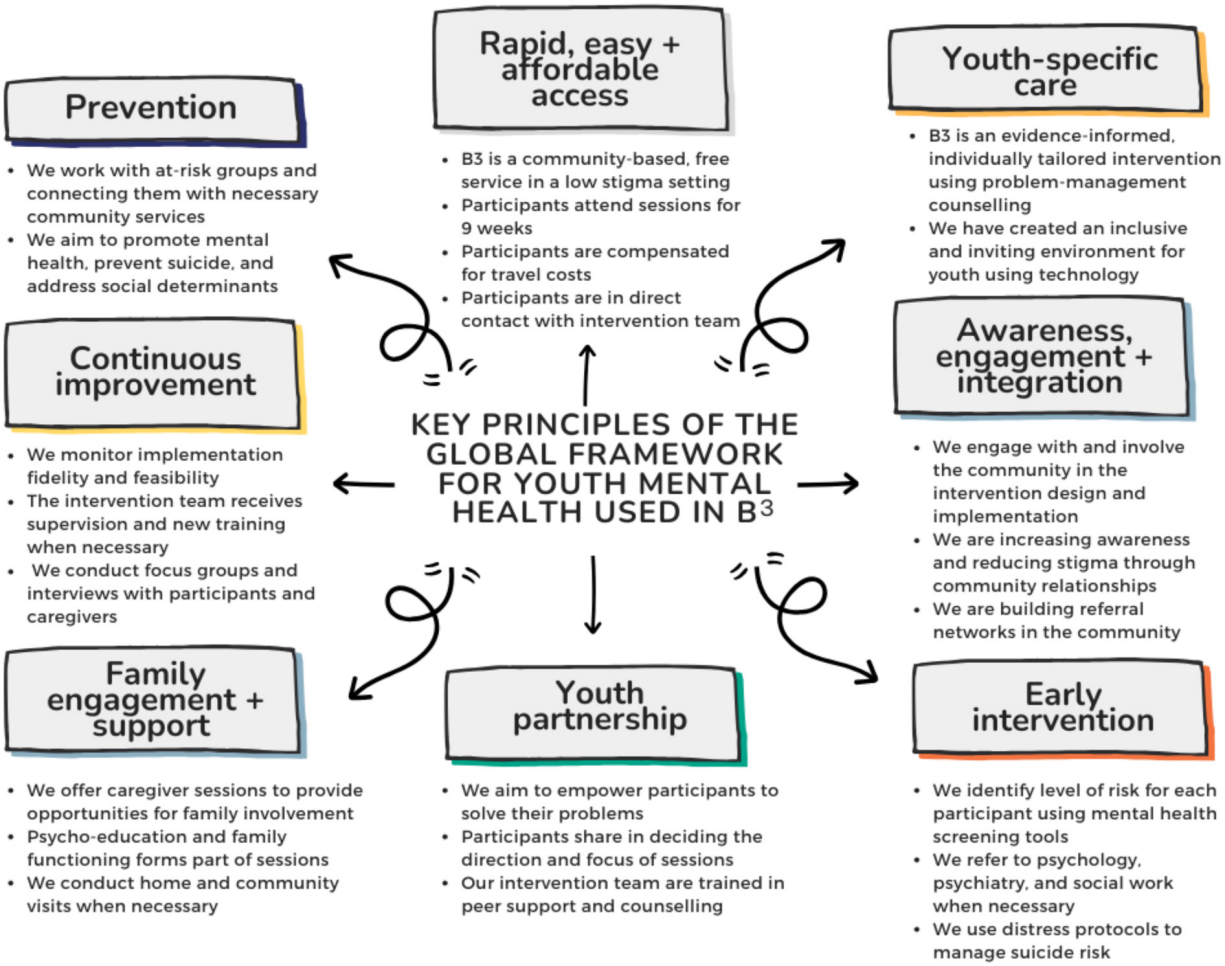
Conceptual model for *Boikoetliso Ba Boko* (B^3^) prototype.

For our B^3^ prototype, we utilized the Global Youth Mental Health framework as the foundation for implementing youth mental healthcare interventions (see Figure 1) (35). This approach ensures that all intervention components are based on efficacy evidence and can provide rapid support to young people, with longer-term referrals to psychiatry, psychology, and social services as needed. In line with the Global Youth Mental Health framework (35), we emphasized three key principles: (i) affordability for sustainable integration into community settings across South Africa, (ii) an appealing physical structure to encourage attendance, and (iii) adequate staffing aligned with the current health service model.

**Figure 2:**
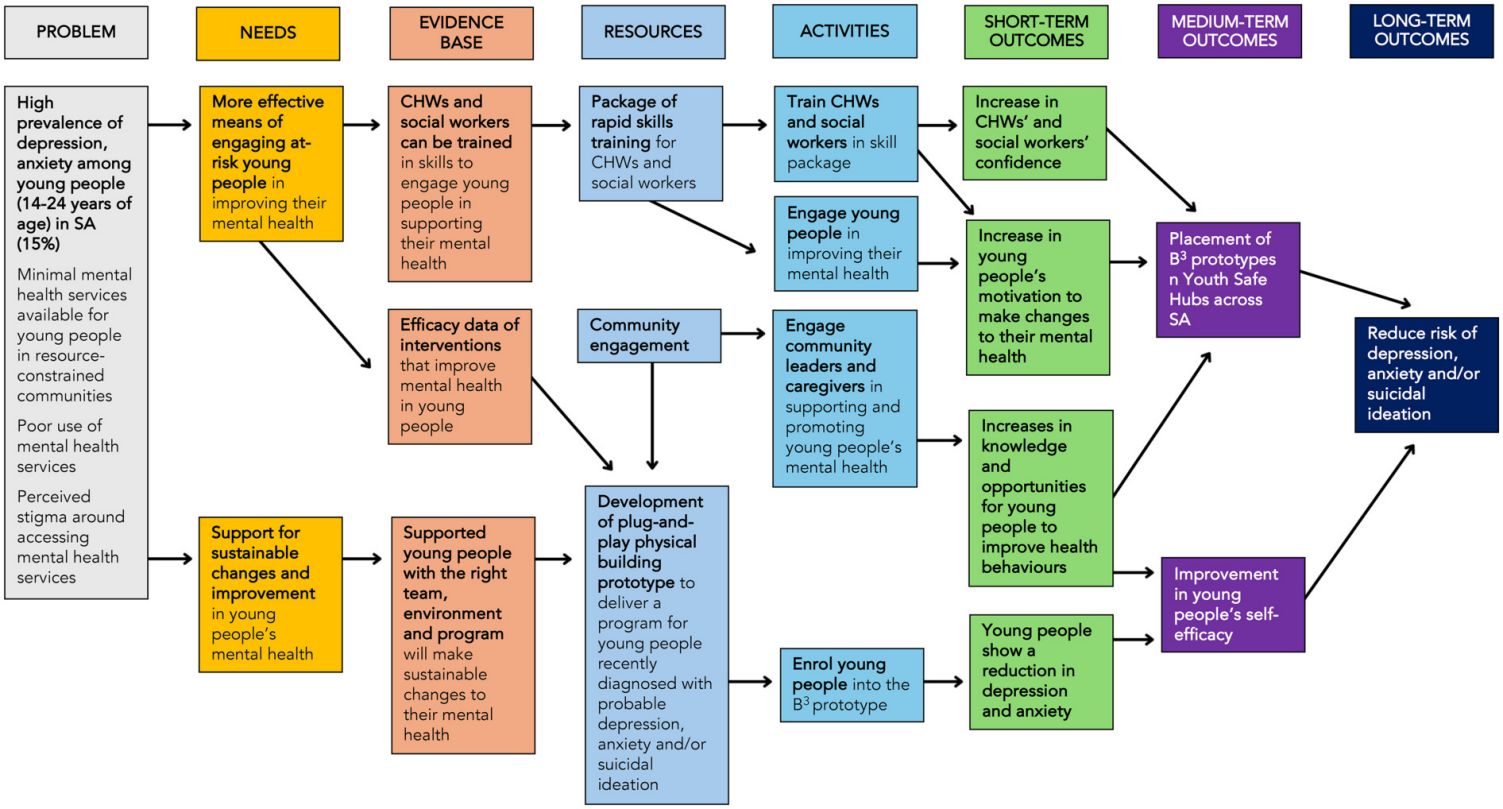
Logic model for *Boikoetliso Ba Boko* (B^3^) prototype.

A Community Advisory Group, consisting of local leaders, community members, caregivers, and health workers, was established to assess the need for youth mental health interventions and identify barriers and facilitators through regular community meetings. Additionally, an Adolescent Advisory Group co-developed the pathways to impact model for the prototype (see Figure 2), contributing to decisions on delivery format, language, and relevant psychosocial norms and needs. Engaging the target population and community early in the process will help to build trust and a sense of co-ownership, which will be essential for the successful and sustainable implementation of the intervention. This engagement will continue throughout the implementation phase to ensure the intervention remains contextually grounded and responsive to local needs. Ongoing community engagement will support continuous adaption and refinement. To further enhance uptake and impact, we will actively foster relationships with youth-serving community organizations and local health services, using these platforms to raise awareness and strengthen community-based support networks.

#### Intervention package

A working group of public health researchers and a community psychologist developed the intervention based on implementation science best practices and insights from the Advisory Groups. WHO Problem Management Plus (PM+) has been identified as an effective intervention for youth mental health and will serve as the foundation of the prototype (36). PM+ is a low-intensity, scalable intervention designed for individuals in resource-constrained communities affected by adversity. It is delivered by non-mental health specialists and involves problem-management counselling along with selected behavioural strategies. The intervention also encourages the involvement of family members and aims to address social support challenges. PM+ emphasises ‘problem management’ rather than ‘problem-solving counselling’, recognizing that young people in these settings often face numerous issues, such as community violence or chronic poverty, that may be difficult to solve or beyond their control (37, 38). The efficacy and flexibility of the PM+ intervention in addressing mental health issues across various contexts and demographics have been well-documented (39–48). In Pakistan, PM+ demonstrated a significant reduction in anxiety and depression by approximately 30% (45, 46) and in Kenya, it showed significant effects on psychological distress (43, 48).

Wellcome Trust's Active Commission identified ‘active ingredients’ for effectively treating and preventing anxiety and depression in young people based on their known efficacy and effectiveness (49). PM+ includes many components recognized as ‘active ingredients’, including collaborative goal setting, problem solving, psychoeducation, and peer support. However, a significant gap in PM+ was noted, particularly regarding the focus on body and brain functions. To address this gap, ‘Brain Gym’ activities were integrated into the intervention. This approach, created by Paul and Gail Dennison, uses physical activity and exercises to enhance focus, concentration, memory, and emotional balance by stimulating neural connections (50). ‘Brain Gym’ movement and coordination activities have been shown to reduce anxiety (51, 52), prevent and treat depression (53, 54), improve sleep quality (55), and enhance general well-being (56, 57). Also, Virtual Reality (VR) has emerged as an innovative tool for supporting individuals with various mental health problems and is particularly appealing to adolescents and young people. VR can improve treatment outcomes through skills training and mindfulness practices, increase access to mental health care by reaching more people and reducing treatment costs, and enhance overall mental well-being across different communities (58–60).

#### Program theory

Implementing interventions with an existing evidence base in new contexts can be more efficient and scalable than developing entirely new interventions (61). The updated Medical Research Council (MRC) and the National Institute for Health Research framework emphasizes the importance of articulating and integrating program theory throughout the development and evaluation of complex interventions (62, 63). The B^3^ prototype integrates cognitive-behavioural, social, and physical mechanisms to address adolescent mental health challenges in resource-constrained settings. B^3^ is grounded in the theory that psychological distress among adolescents can be reduced through the enhancement of problem management skills, emotional regulation, physical self-regulation, and social connectedness. These mechanisms are activated through three evidence-based components: PM+, a low-intensity, scalable WHO-developed intervention that enhances coping through structured problem management, behavioural activation, and support mobilization (36, 39–48); ‘Brain Gym', a set of movement-based exercises aimed at improving cognitive and emotional functioning, reducing anxiety and depression, and promoting overall well-being through physical self-regulation (50–57); and Virtual Reality (VR), which delivers mindfulness, relaxation, and movement activities in an immersive, engaging format to support emotional regulation and reduce psychological distress (58–60).

**Figure 3:**
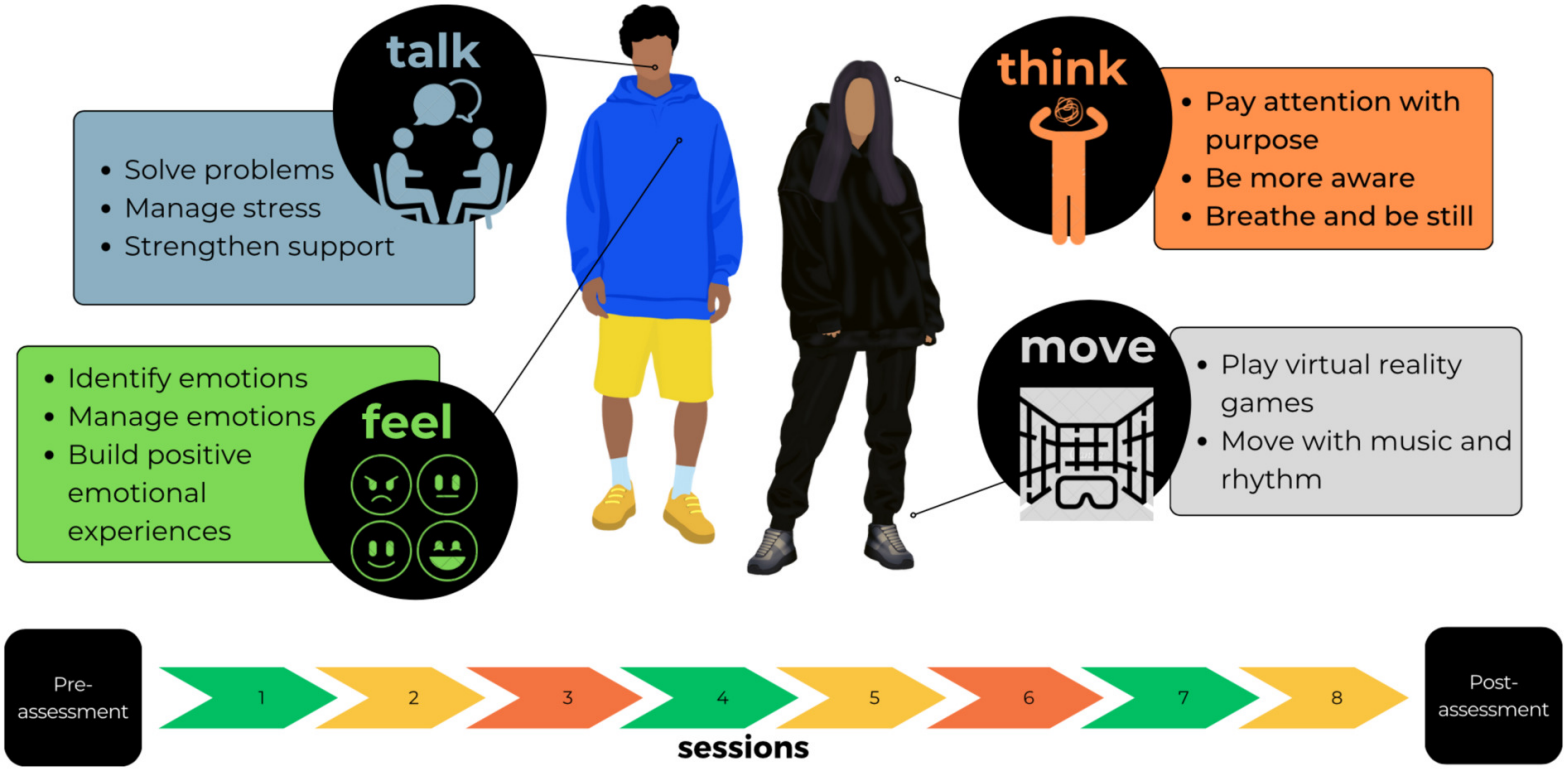
Weekly sessions at *Boikoetliso Ba Boko (B^3^*) prototype.

B^3^ consists of a 9-week program, combining PM+, ‘Brain Gym’ exercises, and VR activities to create a holistic, engaging, and less stigmatized environment. Each week, participants will rotate through four stations (Talk, Think, Feel, and Move), designed to sequentially build skills in emotional regulation, problem management, and physical grounding. Sessions range from 85 to 130 min and are delivered on weekdays and weekends to ensure accessibility (see Figure 3). Talk, Think, Feel, and Move are designed to activate a set of interrelated mechanisms of change. At the cognitive level, the intervention promotes improved problem appraisal, goal setting, and behavioural activation. Emotionally, it supports the regulation of affect through mindfulness practices and physical movement. At the social level, it aims to strengthen support networks through structured peer interactions and engagement with community resources. Finally, on a physical level, the intervention helps regulate physiological arousal and somatic symptoms through movement-based and embodied experiences. These mechanisms are expected to produce outcomes such as reduced emotional distress, improved coping, and increased mental health literacy, which in turn contribute to longer-term outcomes, including enhanced resilience, sustained well-being, and reduced incidence of common mental health conditions.

#### Physical structure

**Figure 4:**
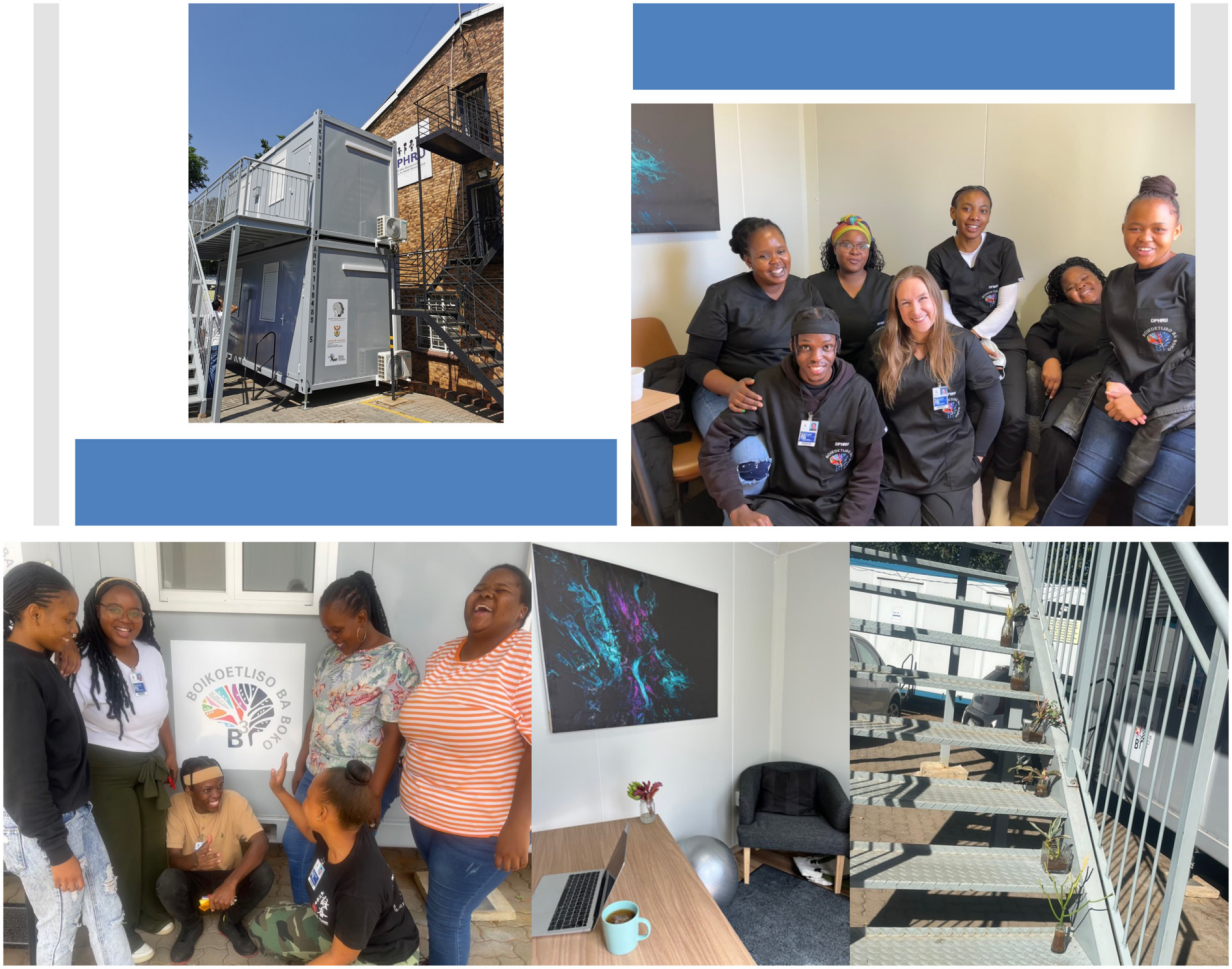
The *Boikoetliso Ba Boko (B^3^*) container.

We designed a two-level building with four rooms, made from shipping containers, featuring built-in electrics, lighting, and air-conditioning (see Figure 4). This ‘plug-and-play’ concept allows for quick, affordable assembly and easy transport. The structure is intended to be integrated into existing community-based spaces across South Africa called Youth Safe Hubs, which offer holistic services and programs to support the social and economic development of young people (64).

#### Staff

To deliver the intervention, a social worker and five certified (national qualification certificate) community health workers (CHWs) will be required. Recruitment of staff will be guided by minimum baseline criteria, including a relevant qualification, at least two years’ experience working with the target population or in a related field, and demonstrated strong interpersonal and reflective practice skills. A 4-week training program, including in-field practice, would be the minimum time required for a psychologist to equip the team to deliver the intervention as outlined in the competency matrix (see Table 2). The team will be trained in an overview of mental health issues, the tenets of the intervention package and activities, distress protocols, self-care practices, and psychological first aid principles. At the end of training, the intervention team will undergo a structured assessment involving observed role-play and reflective discussion to ensure readiness to deliver the intervention. To support the CHWs and social worker, standard operating procedures will ensure uniform delivery of intervention modules. Fidelity to the intervention model will be monitored through regular structured observations using a fidelity checklist, ongoing self-assessment, and monthly supervision by a psychologist. Additional supervision will be available as needed. These quality appraisal and support mechanisms are in place to ensure that the intervention is delivered consistently and effectively.

**Table 2:** Intervention team training in counselling skills.

Counselling skills that can be provided by community health workers	Included in training
Active listening: Teaching CHWs to listen attentively without interrupting, showing empathy, and understanding the participant's concerns and emotions.	✓
Empathy and compassion: CHWs to approach participants with empathy, understanding their emotional and social contexts, and responding in a caring and non-judgmental manner.	✓
Basic mental health support: Providing CHWs with the skills to recognize common mental health issues, offer basic support, and know when to refer clients to professional mental health services.	✓
Motivational interviewing: Training CHWs in motivational interviewing techniques can help them encourage behaviour change in areas like smoking cessation, adherence to medication, and healthy lifestyle choices.	✓
Crisis intervention: CHWs can be equipped with skills to provide immediate support in crises, such as family violence, substance abuse, or suicidal ideation, and to refer to appropriate services.	✓
HIV/AIDS Counselling: Given the high prevalence of HIV/AIDS in South Africa, CHWs should be skilled in pre- and post-test counselling, helping clients understand their HIV status, and providing support for those living with HIV.	x
Health education and promotion: Training CHWs to effectively communicate health information to improve health literacy.	✓
Cultural competence: Ensuring that CHWs understand and respect the cultural beliefs and practices of the communities they serve, allowing them to deliver care that is culturally sensitive.	✓
Problem-solving techniques: Helping CHWs learn how to guide clients through a structured approach to solving personal or health-related problems.	✓
Referral skills: Training on how to recognise cases that need more specialized care and effectively refer clients to appropriate healthcare professionals or services.	✓
Confidentiality and ethics: Emphasizing the importance of maintaining confidentiality and adhering to ethical standards in all interactions with participants.	✓
Stress management: Teaching CHWs how to manage their stress and avoid burnout, which is crucial given the challenging environments in which they work.	✓

### Data collection

Participants will serve as their own controls, with the primary outcome being the change in depression or anxiety status from baseline to exit after the 9 sessions. Assuming a 20% reduction in depression or anxiety (Cohen's d = 0.2), a sample size of 200 will provide 80% power. Data will be collected by the CHWs using secure tablets and the REDCap data collection platform will ensure real-time capturing, reporting and quality control (65). This study may provide early indications of the intervention's potential impact, helping to refine hypotheses for the efficacy trial. It will also evaluate whether the assessment measures are valid, reliable, and sensitive to this target population and context. Table 3 outlines the specific implementation data to be collected.

**Table 3:** Implementation data collection.

Domain	Measure	Assessment time point		
		**Pre-assessment** (Before session 1)	**During intervention** (Session 1–Session 8)	**Post-assessment** (After session 8)
Demography	Sociodemographic questionnaire (date of birth; gender; language; relationship status; education; occupation; household assets; food insecurity)	✓	x	x
Medical	Medical questionnaire (prior diagnosis of anxiety, depression, tuberculosis, epilepsy, HIV; on medication for anxiety, depression, tuberculosis, epilepsy, HIV)	✓	x	✓
	WHO functional Impairments Questionnaire (36)	✓	x	x
	Blood pressure	✓	x	✓
Daily functioning	WHO disability assessment schedule 2.0 (71)	✓	x	✓
Tobacco, alcohol, and drug use	Tobacco use questionnaire (72); AUDIT-Questionnaire (73); drug use questionnaire	✓	x	✓
Stressful life events	Adverse childhood experiences questionnaire (74)	✓	x	✓
Anxiety	GAD-7 (30)	✓	x	✓
Depression	PHQ-9 (29)	✓	x	✓
Self-efficacy	Generalized self-efficacy scale (75)	✓	x	✓
Social support	Social support questionnaire	✓	x	✓
Sleep	Pittsburgh sleep quality index (76)	✓	x	✓
Psychological well-being	PSYCHLOPS (77)	✓	✓	✓
Suicide risk	WHO thoughts of suicide questionnaire (36)	✓	✓	✓

### Process evaluation

**Figure 5:**
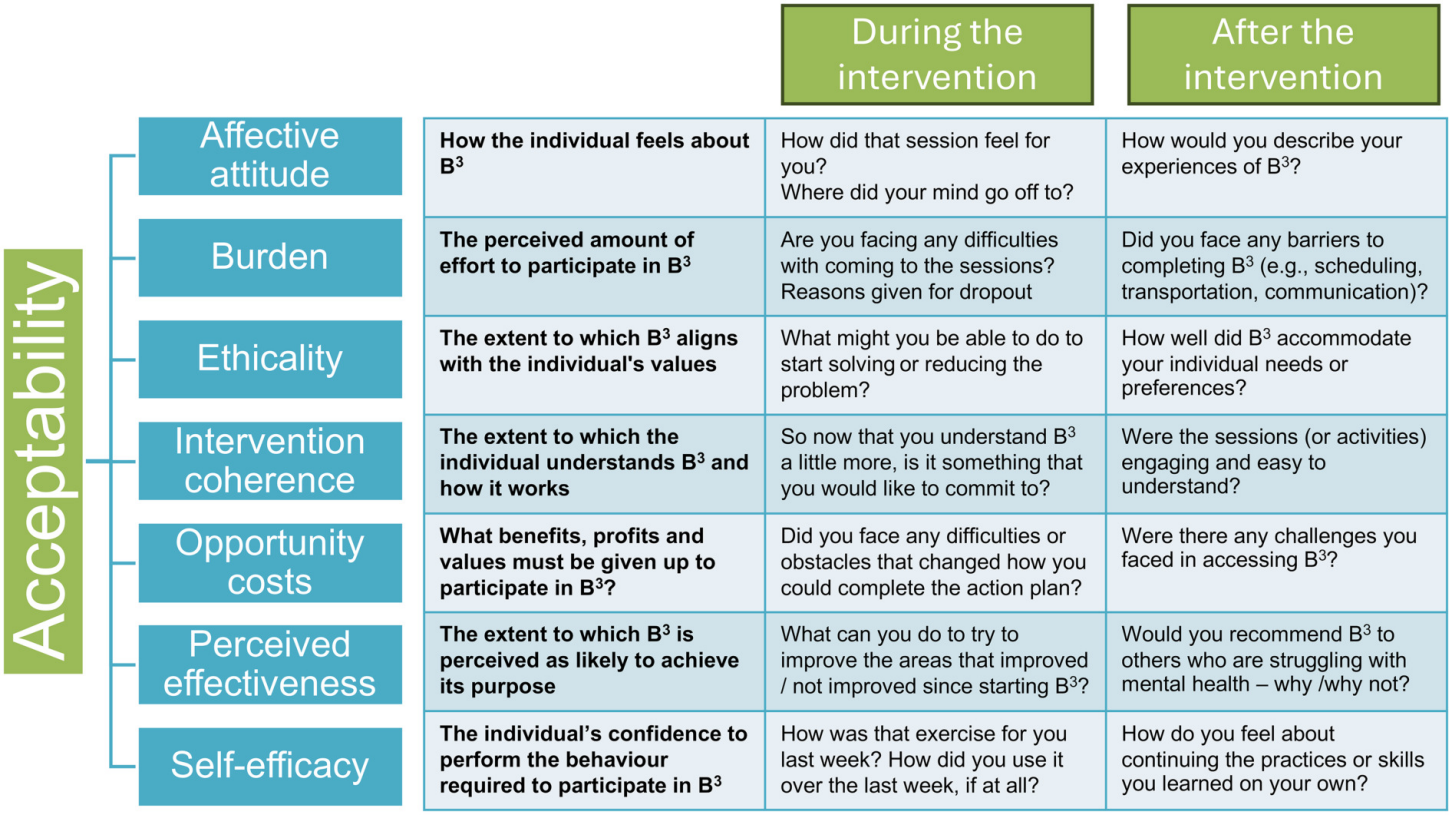
Acceptability model for *Boikoetliso Ba Boko (B^3^*) prototype.

Following MRC guidance on process evaluation (61, 62), we will assess implementation feasibility, mechanisms of impact, and context. Implementation feasibility assessment will focus on what is delivered and how, with compliance monitored via logs and discussions, to determine whether recruitment, retention, and implementation strategies are effective in this setting. Fidelity will be evaluated through observations of CHWs during one-on-one sessions by the social worker, while intervention dose will be tracked using diaries to record contact frequency and duration. Process indicators, including recruitment and retention, and indicators of program delivery will also be assessed. To understand the intervention's impact and how it meets the mental health needs of adolescents and young people, the acceptability of the intervention will be explored by focusing on the participants’ experiences. In-depth interviews and focus groups will be conducted with participants, caregivers, and the CHWs delivering the intervention, asking questions based on the seven component constructs of acceptability (66), as detailed in Figure 5 below. In assessing the context, we will aim to identify factors that might act as barriers or facilitators to intervention implementation or effects. These qualitative methods (interviews and focus groups) will be used to further explore specific issues of relevance, in terms of participants’ experiences of B^3^, key issues facing participants and factors influencing implementation.

### Data management and analysis

REDCap will be used for data management and storage to ensure the confidentiality, security, and integrity of the data collected (65). All information collected will remain confidential and will be stored securely at the University of the Witwatersrand and the Developmental Pathways for Health Research Unit, meeting international security and safety standards. Strict security measures including access controls, computer security, data encryption techniques, confidentiality agreements, and staff training are in place. The results of the research will be published and shared during scientific meetings, as well as with policy and practice stakeholders. Any information that identifies people will not be published or made public by the researchers. All data will be presented as group data, rather than individual data.

Descriptive statistics will be used to summarize markers of feasibility, such as fidelity, dose, and reach. Variations between participants in terms of factors such as fidelity and reach will be analyzed at different points in time using detailed modelling. The transcriptions of focus groups will be imported into MaxQDA coding software, where independent, trained research assistants will code, group, and review themes and sub-themes to identify key findings that emerge over the course of the intervention. The quantitative and qualitative analyses will not be used in isolation. Rather, in-depth qualitative data from participants, caregivers, and the intervention team will be triangulated with quantitative monitoring data documenting the delivery of intervention components. Qualitative data will be analyzed thematically using Braun and Clarke's six-phase framework (67), allowing for both inductive and deductive coding aligned with the study's objectives. To ensure robust triangulation, we will adopt a convergent design by comparing themes across data sources and methods to identify convergence, divergence, and complementarity. This process will be guided by established triangulation protocols in qualitative research (68) and principles for managing multiple qualitative data sources (69), thereby enhancing the credibility and depth of the findings.

### Economics

In resource-constrained settings, cost data is crucial for understanding the feasibility of scaling up interventions (5). In this study, cost data will be collected to determine the total cost of designing, starting up, and implementing the prototype. This will include direct costs such as staff time, training, materials, facilities, and operational expenses, captured through financial records and staff time sheets. While we acknowledge that there is no universally accepted method for evaluating cost-effectiveness in such contexts (70), the primary aim of this economic component is to collate cost data that can inform future cost evaluations and guide resource allocation. Rather than assessing cost-effectiveness or economic feasibility directly, we will focus on generating comprehensive cost information that could support future analyses and decision-making around the scalability and sustainability of the intervention.

## Discussion

The development and implementation of B^3^ offers a proactive response to the mental health needs of adolescents and young people, particularly in resource-constrained settings. By adapting evidence-based interventions such as PM+ and integrating innovative components like ‘Brain Gym’ activities and Virtual Reality, the B^3^ prototype aims to provide a holistic and engaging mental health intervention that is based in the community and links to the existing public health system. Grounded in the Global Youth Mental Health framework (35) and including the Wellcome Trust's Active Commission ‘active ingredients’ for effectively treating and preventing anxiety and depression in young people (49), the B^3^ prototype aligns with established best practices, as well as ensures that the intervention is tailored to the specific developmental and contextual needs of adolescents and young people. The inclusion of evidence-based and innovative components within the guidelines of recognized frameworks enhances the credibility and potential impact of B^3^ prototype.

A major strength of this study is our community-driven approach, which involves both a Community Advisory Group and an Adolescent Advisory Group. This collaboration ensures that the intervention is culturally relevant and addresses the unique challenges faced by adolescents and young people. Furthermore, the stigma and treatment gaps that persist in many communities can be addressed by promoting awareness of mental health issues and showcasing effective community-based interventions. The promising evidence from prior adaptations of community-based interventions, including the YouFB in Zimbabwe (25–27), reinforces the potential efficacy of the B^3^ prototype in providing mental health care to adolescents and young people experiencing anxiety and depression, and suicidal ideation.

As a Phase I implementation study, we aim to monitor the impact of the B^3^ prototype rigorously and adaptively throughout its development and implementation. The combination of qualitative and quantitative evaluation methods will provide a comprehensive understanding of the intervention's effectiveness and feasibility. Additionally, this study will monitor the costs associated with the intervention, which is particularly important in resource-constrained settings. By collecting detailed cost data on design, implementation, and delivery, we aim to inform future assessments of economic feasibility and support decision-making regarding the potential for scale-up in similar contexts.

The findings from this study will play a crucial role in informing the development of a Phase II randomized controlled trial protocol aimed at assessing the efficacy, scalability, and sustainability of the B^3^ prototype. Insights gained from this Phase I implementation study can be used to refine the prototype based on participant feedback and data analysis, highlighting how specific components contribute to its overall effectiveness. This feasibility study will also identify potential risks and barriers, ensuring that the larger efficacy trial is both ethically and practically sound. By identifying practical and logistical challenges, such as staffing, technology access, and funding, early on, the study will help prevent costly mistakes in the full-scale trial and allow for more efficient planning. Demonstrating feasibility and acceptability increases the likelihood of securing funding approval and ethical clearance for the larger trial, offering evidence of the intervention's potential for success. Ultimately, fostering long-term resilience and well-being among adolescents and young people requires systemic changes in mental health care, with interventions like the B^3^ prototype leading the way toward more inclusive and responsive services in resource-constrained communities.
